# Measuring financial satisfaction of Indonesian young adults: a SEM-PLS analysis

**DOI:** 10.1186/s13731-023-00281-4

**Published:** 2023-03-17

**Authors:** Farizka Shafa Nabila, Mahendra Fakhri, Mahir Pradana, Budi Rustandi Kartawinata, Anita Silvianita

**Affiliations:** School of Communication and Business, Telkom University, Jl. Telekomunikasi No. 1, Buah Batu, Bandung, Indonesia 40257

**Keywords:** Financial attitude, Financial management, Financial satisfaction

## Abstract

People in Indonesia, particularly members of Generation Z, frequently struggle to manage their financial situation both now and in the future. The problem is brought on by a lack of understanding of financial investments. The purpose of this study is to ascertain the financial standing of Generation Z. A questionnaire with 100 respondents was employed in this investigation. In this study, financial attitudes serve as the independent variable, financial management serves as the intervention variable, and financial satisfaction serves as the dependent variable. A Likert scale was utilized as the measurement in the quantitative research technique. In this work, structural equation modeling (SEM) and SmartPLS software were utilized to process the data. The financial attitude variable has a positive and significant impact on financial happiness that is mediated by financial management. We also offer some recommendations and future research directions related to this topic.

## Introduction

According to population, Indonesia is the fourth-largest nation on earth. Annur, (2020) estimates that there are already 274 million people living in Indonesia. According to data from a survey conducted by the Financial Services Authority (OJK), only 12.6% of Indonesia's population has good financial planning (Annur, 2020). According to IPSOS 2017 data (Primadhyta, 2017), more than 70% of Indonesian parents rely on their monthly income for their children's education and expenses, and as many as 86% of parents are willing to give up their retirement funds to meet their children's educational needs. Nearly a quarter of them acknowledge that they do not have a point of reference for how many education dollars need to be budgeted.

There are five generations, and they are categorized based on sociocultural aspects like historical events as well as demographic information like birth year. The way financial asset is managed varies from generation to generation. The first generation is that of the baby boomers, who were born between 1943 and 1960. This group saves for their children's inheritance while also splurging on houses, land, and vehicles. The second group consists of people born between 1961 and 1981, known as Generation X. This generation is approaching retirement and invests in real estate. The members of Generation Y, those born between 1982 and 1994, use the internet to do business and start making mortgage payments and always want to look stylish. The fourth generation, Generation Z, who grew up with technology and prioritizes social media fame, was born between 1995 and 2010. The fifth generation, Generation A (alpha), was born between 2011 and the present and relies on technology and devices to access information from numerous sources (Dimock, 2019).

The Millennial Generation makes up 25.87 percent of Indonesia's population, while Generation Z makes up 27.94 percent, according to the Central Statistics Agency's 2020 Population Census (Shalihah, 2021). Generation Z in Indonesia is recognized to differ significantly from older generations in a number of ways. Generation Z is the term used to describe the boundary-less generation (Rakhmah, 2021). According to Jenkins (2017) in his essay "Four Reasons Generation Z will be the Most Different Generation", Generation Z has different aspirations, interests, and career perspectives and has an impact on most people's culture and attitudes (Kartawinata et al., 2021).

Saving, having a career, managing funds, investing, and other financial activities are all priorities for Generation Z. These are ranked according to how urgently they require something. The priorities of Generation Z are listed below, according to a Charles Schwan survey from February 2019 (Refsi, 2021). Saving is the top priority for young adults since they are worried about their financial situation in the future. Working is one strategy used by Generation Z to lessen concerns about their financial situation. Additionally, according to a survey by Raddon Research, 77% of people between the ages of 14 and 22 had worked freelancing or part-time and received benefits. The remaining 38% of them, on the other hand, intend to work after finishing their studies. Up to 71% of Generation Z feel quite anxious about their financial situation (Ozkan & Solmaz, 2015). By securing employment that will help them reach their goals, they establish their financial plans for the future in order to prevent this circumstance (Lusardi, 2019). Generation Z investment is given the lowest priority compared to other priorities since they are still unfamiliar with financial investment tools.

Well-being is the end goal of all elements of life. The definition of well-being is the state of being at ease, healthy, and content (Darmawan & Pamungkas, 2019). Financial well-being is one of the dimensions of well-being, which is a feeling of satisfaction of people with their financial situation, improvement in living standards, ability to meet needs, safety, comfort, and well-being, in this case satisfied with monthly income, in both material and non-material aspects (Taft, et al., 2013). Good financial management can lead to welfare; as such, everyone should set goals for their financial future, safeguard and grow their wealth, manage their cash flow (income and spending), and handle any financial risks (Andani, 2018).

To achieve financial contentment is the goal of financial management. Financial satisfaction is seen as a subjective indicator of a person's level of contentment with his financial situation and a measure of that person's financial well-being, according to (D'Agostino, et al., 2020). The amount of income, the nature of one's employment, and age are additional elements that influence financial satisfaction. Additionally, one's financial mindset and money management skills have an impact on their level of financial contentment (Owusu, 2021).

Financial attitude is a person's viewpoint on money management, including budgeting, setting aside money for emergencies, and predicting future financial conditions (Firli & Hidayati, 2021). The Financial Services Authority (OJK) goes on to say that it helps the general public acquire a financial mindset so they can set financial objectives and conduct financial planning. According to prior research, there is a correlation between financial attitude and financial satisfaction, which means that those with a positive attitude about money will have an impact on someone else's level of financial satisfaction because it is so simple to access information on current issues (Darmawan & Pamungkas, 2019).

Financial management is evaluated as a description of a person's financial decision-making process and ability to prioritize his requirements and wants (Loke, 2017). It backs up the study done by Owusu (2021), which found that having strong financial management had a beneficial impact on financial contentment. Therefore, the degree of financial satisfaction is influenced by those who practice appropriate money management, including timely bill payment, price comparison when making an installment or credit purchase, and future financial planning. There has been extensive research on the factors affecting financial contentment (Lusardi, 2019; Xiao & Porto, 2017). However, financial management's mediating impact on financial attitude and financial contentment has not been explored extensively. Due to Generation Z's impending entry into the workforce and growing awareness of personal management, we feel compelled to perform this research with an emphasis on Indonesian young adults.

## Literature review

### The concept of financial satisfaction

The attainment of one's life goals, good physical and mental health, and education all contribute to one's quality of life (Neugarten, et al., 1961). Since life satisfaction is regarded as the most important aspect of quality of life, every person strives to raise their standard of living (Pradana et al., 2022). The state of one's financial satisfaction is also a major factor in determining one's level of life satisfaction and quality (Owusu, 2021). The satisfaction people feel in relation to their current financial situation is described as financial contentment by (Atlas, et al., 2019). A subjective assessment of the degree to which financial resources can meet present and future requirements is known as financial contentment (Xiao & O’Neill, 2018). Within the broad framework of consumer and family economics, a framework is required to explain and forecast personal financial satisfaction (Joo & Grable, 2004). Few attempts have been made to describe, provide, and test a multidimensional framework of financial contentment, despite the fact that empirical work has been done to construct and assess financial satisfaction (Kartawinata et al., 2021). A conceptual framework of the determinants of financial satisfaction, according to researchers and academics, is required because, with a better understanding of the variables affecting financial satisfaction, professionals in the family and consumer sciences can employ more effective strategies to improve people's quality of life (Joo & Grable, 2004).

### The concept of financial attitude

A person's mentality, viewpoints, and opinions on their finances are referred to as their financial attitude. Obsession, power, effort, insufficiency, retention, and security are the six concepts that make up financial attitude (Wibowo & Dewi, 2021). Individual behavior in financial management, financial planning, and investment decisions can all be influenced by one's attitude toward money. Financial attitude is a way of thinking about managing money that is influenced by one's ideas, emotions, and behavior. It can have an impact on how one borrows money and how they decide whether or not to make an investment (Siswanti & Halida, 2020). Consumption, saving, investing, and retirement planning are the first four steps in developing a positive financial mindset (Zahriyan, 2016).

Orientation to personal finance, debt philosophy, financial stability, and personal financial assessment are other factors that might be included while evaluating financial attitude (Ibrahim & Alqaydi, 2013). According to Joo and Grable (2004), financial attitude is a psychological trait that manifests itself through the evaluation of a specific entity with varying degrees of favor or disfavor. According to a widely accepted definition, one's financial attitude refers to their agreement or disagreement with current financial situations. The greater one's financial attitude, the more responsibility that person will assume while managing their finances (Darmawan & Pamungkas, 2019).

Financial attitude and financial contentment are significantly correlated, with a favorable financial attitude enhancing financial satisfaction (Arifin, 2018). Additionally, a study by Herdjiono and Damanik (2016) found a connection between financial mentality and money management. How one manages their money is significantly influenced by their financial thinking. If one has good financial management, they will be more knowledgeable while making financial management decisions (Ali et al., 2015).

Based on the description above, we construct hypotheses 1, 2, and 3, respectively:

Hypothesis 1 (H1). There is an influence of financial attitude on financial management in Generation Z.

Hypothesis 2 (H2). There is an influence of financial attitude on financial satisfaction in Generation Z.

Hypothesis 3 (H3). There is an indirect effect of financial attitude on financial satisfaction through financial management in Generation Z.

### The concept of financial management

**Fig. 1:**
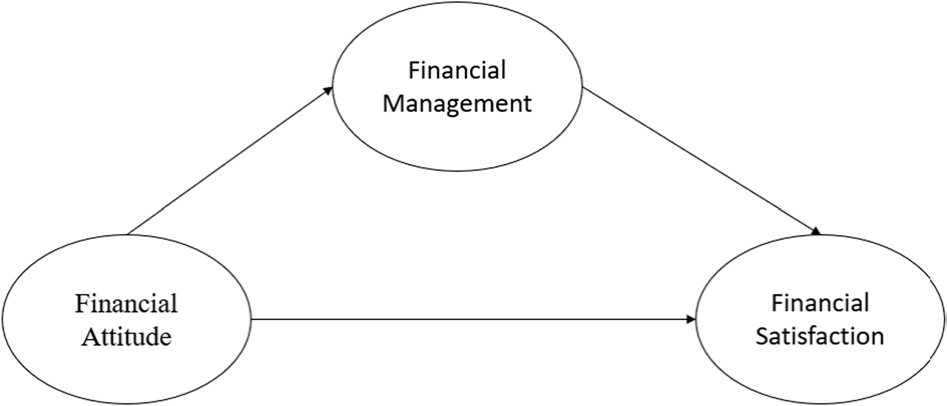
Research model

Financial management is the ability to produce sound finances, make judgments using excellent financial management techniques, and develop financial management abilities (Owusu, 2021). Financial management is a set of actions that involves cash, credit, investments, insurance, and pension finance strategy, implementation, and evaluation (Mien & Thao, 2015). Financial management, on the other hand, is a process used to generate income with expenses that are kept to a minimum and manage them successfully to reach goals (Sujarweni, 2017). Due to the impact of a person's desire to meet their requirements through income, financial management is necessary (Lusardi, 2019) (Fig. 1).

Financial management and financial contentment have a substantial association, according to some prior studies, where the better one's financial management, the higher one's level of satisfaction with their financial situation will be (Ali et al., 2015; Kaiser et al., 2022). If a person has better financial management, he or she will be able to budget effectively, buy what they want, and lay money aside for investments, which will lead to high financial happiness (Arifin, 2018).

Based on the description above, we proposed Hypothesis 4.

Hypothesis 4 (H4). There is an influence of financial management on financial satisfaction in Generation Z.

## Methodology

### Research design

This investigation utilizes a quantitative methodology. Quantitative analysis demonstrates how one variable influences other others (Creswell, 2012). A Google Form that is available online was utilized to collect the data for the study. The interval scale was employed as the measurement scale in this study for variables that are both dependent and independent. For quantitative analysis, respondents were given five options to choose from for each variable on a scale from 1 (Strongly Disagree) to 5 (Strongly Agree).

### Sample and data collection

All members of Generation Z in Bandung, West Java, were included in the study's sample. Due to their vast number starting to enter the workforce and lack of knowledge of personal financial management, Generation Z is seen as being more suitable in this study. Additionally, respondents are told that the information they supply will be kept anonymous, and participation in the study is entirely optional. In October 2021, this questionnaire was made available. According to Table 1, the sample for this study was predominately female, with 71% of respondents being female. The majority of respondents are between the ages of 18 and 20, and 89% are students. Additionally, the majority of those surveyed earn less than IDR 1,000,000.

**Table 1:** Socio-demographic characteristics of the respondents

No	Demographic characteristics	Frequency	Percentage
1	*Gender*		
	Male	29	29%
	Female	71	71%
2	*Age*		
	18–20 years	80	80%
	20–23 years	7	7%
	23–25 years	13	13%
3	*Employment status*		
	Student	89	89%
	Employees	9	9%
	Entrepreneur	2	2%
4	*Monthly income*		
	< Rp 1.000.000,-	44	44%
	Rp 1.000.001.- to Rp 3.000.000,-	40	40%
	Rp 3.000.001,- to Rp 5.000.000,-	13	13%
	> Rp 5.000.000,-	3	3%

Source: Author’s result (2021)

### Data analysis

One of the statistical methods used in SEM (structural equation modeling) is called partial least square (PLS), which aims to evaluate the predictive relationship between constructs by assessing whether or not there is a link (Abdillah & Hartono, 2015). Versions of PLS that simultaneously test the measurement and structural measurement models can be used. PLS is used to predict how variable X will affect variable Y and to clarify the theoretical connection between the two variables. On the other hand, OLS (ordinary least squares) regression assumptions such as that the data must be normally distributed multivariate and that there is no multicollinearity issue between exogenous variables are eliminated in SmartPLS 3.0 (Ramayah, et al., 2018).

## Results and discussion

The validity test reveals a correlation with the concept of evaluating a concept. According to Chin (1995) in Abdillah and Hartono, (2015), the values of outer loading > 0.7, communality > 0.5, and average variance extracted (AVE) > 0.5 are the main guidelines utilized in testing convergent validity. In this study, the internal consistency of the measurement tool was assessed using a reliability test. For the PLS reliability test, there are two benchmarks available: Cronbach's alpha and composite reliability from indicator blocks that measure structures (Ramayah, et al., 2018). Hair et al. (2008) state that although a value of 0.6 is still acceptable, the composite reliability or rule-of-thumb alpha value must be better than 0.7 in (Abdillah & Hartono, 2015). The following outcomes were obtained from the reliability and validity test using the SmartPLS software:

A requirement for having strong reliability is that the output results of Cronbach's alpha or composite reliability values for each must be more than 0.7. Therefore, it can be concluded that the data are reliable and all variables have a high level of reliability because the three latent variables (financial attitude, financial management, and financial happiness) have CA and CR values more than 0.7. Table 2 demonstrates that each of the three variables—financial attitude, financial management, and financial satisfaction—has an AVE value greater than the required threshold of 0.5, indicating that the validity requirements have been met.

**Table 2:** Evaluation of measurement model

Factors	Cronbach's alpha	Composite reliability	Average variance extracted (AVE)
Financial attitude	0.895	0.915	0.577
Financial management	0.815	0.868	0.526
Financial satisfaction	0.910	0.927	0.613

Source: Author’s result (2021)

If the outer loading indicator is more than 0, convergence validity is considered to be good (Ghozali & Latan, 2015). However, if the outer loading value is discovered to be in the range of 0.5–0.6, it is deemed sufficient to satisfy the convergent validity criterion. When doing the validity test, it is necessary to exclude any indicators that do not meet the criteria, such as those with an outer loading value of less than 0.5, which denotes that they are not significant (Ramayah, et al., 2018). Table 3 shows the results of outer loading test.

**Table 3:** Outer loading

Variable	Indicator	Questions	Outer loading	Conclusion
Financial attitude	FA1	Financial planning is important to me	0.793	Valid
	FA2	Having a financial plan helps me to make decisions about financial investments	0.784	Valid
	FA3	I am aware of the importance of financial investment	0.820	Valid
	FA4	Thinking about where I will be financially in the next 5 or 10 years is very important to ensure the financial condition	0.776	Valid
	FA5	If I have money, I prefer financial investment to ensure a life in the future compared to spending it	0.718	Valid
	FA6	I need to prioritize primary needs	0.593	Valid
	FA7	I need to plan for the negative possibilities of my income (for example loss of income during the Covid-19 pandemic	0.850	Valid
	FA8	One of the benefits of financial planning is to prevent debt bondage	0.713	Valid
Financial management	FM1	I always pay bills before the due date	0.609	Valid
	FM2	I always read the detail of my bills to compare monthly usage or rate changes	0.668	Valid
	FM3	I keep tract of monthly expenses	0.653	Valid
	FM4	I usually budget monthly expenses	0.795	Valid
	FM5	I have some money saved for emergencies	0.858	Valid
	FM6	I allocate part of my income to financial investment	0.739	Valid
Financial satisfaction	FS2	I am satisfied because the amount of my income receive can meet my needs	0.789	Valid
	FS3	I am satisfied because the amount of saving I currently have can meet my need	0.803	Valid
	FS4	I am satisfied because I can Pay my bills on time	0.695	Valid
	FS5	I am satisfied because I can manage my current income and expenditure budget	0.800	Valid
	FS6	I am satisfied because I can buy the things I want	0.792	Valid
	FS7	I am satisfied that I have a long-term financial investment	0.825	Valid
	FS8	If I had a loss of income, I could manage for a period of time (e.g., for 3 months)	0.758	Valid
	FS1	I am satisfied with my current financial situation	0.793	Valid

Source: Author’s Result (2021)

There is not an exterior loading indicator with a value of 0.5, as shown in Table 3. This indicates that the indicator has been approved for research usage and is usable for additional analysis. The cross-loading with its construct was used to measure the discriminant validity test. If the cross-loading indicator's value on a given variable is higher than that of other variables, the indicator is said to have discriminant validity. The results of the cross-loading factor utilizing the SmartPLS software can be seen in Table 4.

**Table 4:** Discriminant validity

Indicator	Financial attitude	Financial management	Financial satisfaction
FA1	0.793	0.390	0.352
FA2	0.784	0.467	0.376
FA3	0.820	0.484	0.408
FA4	0.776	0.435	0.408
FA5	0.718	0.647	0.488
FA6	0.593	0.257	0.186
FA7	0.850	0.644	0.492
FA8	0.713	0.377	0.390
FM1	0.517	0.609	0.455
FM2	0.424	0.668	0.429
FM3	0.439	0.653	0.413
FM4	0.413	0.795	0.653
FM5	0.552	0.858	0.535
FM6	0.436	0.739	0.424
FS2	0.264	0.390	0.789
FS3	0.349	0.522	0.803
FS4	0.483	0.379	0.695
FS5	0.498	0.563	0.800
FS6	0.413	0.568	0.792
FS7	0.468	0.669	0.825
FS8	0.449	0.558	0.758
FS1	0.361	0.496	0.793

Source: Author’s result (2021)

**Fig. 2:**
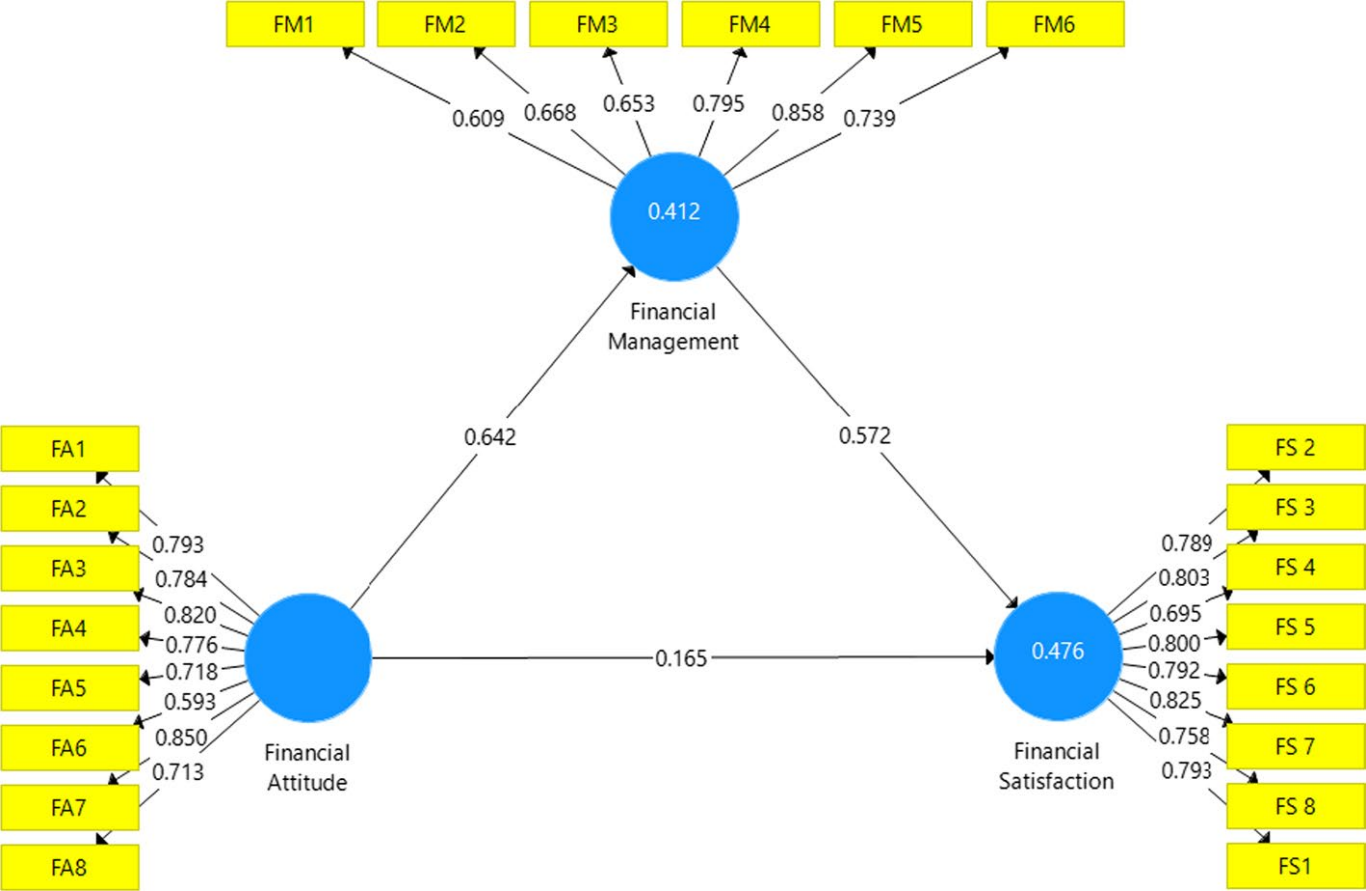
Output bootstrapping. Source: Author’s result (2021)

When compared to the cross-loading values on other variables, Table 4 demonstrates that it satisfies the cross-loading requirements where each variable has the biggest cross-loading value on the variables it forms. Thus, the markers used in this study can be said to have strong discriminant validity (Fig. 2).

- **Evaluation of structural model**
  By comparing the path coefficient value in the structural model with the R-value for the dependent construct, the measurement of the structural model (Inner Model) in PLS is assessed (Ghozali & Latan, 2015). The value of R-square is the coefficient of determination on the endogenous construct. The higher the R-square value, the better the prediction model of the proposed research model (Abdillah & Hartono, 2015).

We see from Table 5 that the R-square values for the financial management variable and the financial happiness variable, respectively, are 0.412 and 0.476. The financial attitude variable's R-square value has a 0.412 effect on financial management, with the remaining 0.588 being influenced by other variables outside of this study. Based on this, the indicators utilized in this study can only account for 41.2% of the variance as a financial management element, with other variables accounting for the remaining 58.8%.

**Table 5:** R-square

Variable	R-square
Financial management	0.412
Financial satisfaction	0.476

Source: Author’s result (2021)

The satisfaction variable's R-square value is 0.476. This implies that the contribution of the financial happiness variable is 0.476, with external factors accounting for the remaining 0.524. As a result, only 47.6% of financial satisfaction can be explained by the variables used in this study; the remaining 52.4% must be accounted by other variables.

b. **Hypothesis test result**
  You can examine the t-statistic value between the independent variable and the dependent variable to determine the significance of the predictive model in testing the structural model. The t-statistic between the independent and dependent variables in the path coefficient table in the SmartPLS output can be used to determine the importance of the predictive model in evaluating the structural model (Table 6).

**Table 6:** Hypothesis test

Variable	Original sample	Sample mean	Standard deviation (STDEV)	T-statistics (|O/STDEV|)	P values
Financial attitude—> financial management	0.642	0.658	0.052	12.285	0.000
Financial attitude—> financial satisfaction	0.165	0.161	0.123	1.344	0.180
Financial management—> financial satisfaction	0.572	0.582	0.113	5.042	0.000
Financial attitude—> financial satisfaction mediated by financial management	0.367	0.384	0.086	4.268	0.000

Source: Author’s result (2021)

### Financial attitude's effect on financial management

The path coefficients table indicates that there is a positive influence on financial management because the path coefficients value is positive 0.642. The *p* value of 0.000 is less than 0.05 and the *t*-statistic of 12,285 is larger than 1.96, indicating a significant effect. In other words, the financial attitude variable significantly and favorably affects financial management. So, the idea that financial attitude has an impact on financial management is acknowledged.

### Financial attitude's effect on financial satisfaction

The path coefficient value is positive 0.165, which indicates that there is a positive influence on financial happiness, according to the route coefficient table above. The results then have t-statistics of 1.344 1.96 and *p* values of 0.180 > 0.05, demonstrating that they do not fit the criteria. Therefore, it can be said that having a favorable financial attitude has a small but positive impact on financial contentment.

### Financial management's effect on financial satisfaction

The route coefficient value is positive 0.572, which indicates a favorable influence on financial happiness, according to the path coefficient table. Then, it has a t-statistic of 5.042, which indicates a significant effect because the value is more than 1.96 and the *p*-value is less than 0.05. Additionally, it is evident that the financial management component significantly and favorably affects financial contentment. So, it is accepted that financial management has an impact on financial contentment.

### Financial management mediates the impact of financial attitude on financial satisfaction

Based on the preceding table, it can be inferred that there is an indirect influence between financial attitude variables on financial satisfaction through financial management or that there is a mediating effect between financial attitude and financial satisfaction. The route coefficient value was discovered to be positive 0.367, indicating a favorable influence on financial contentment. Additionally, it has a t-statistics value of 4.268, which indicates that the link between the influence of financial attitude on financial satisfaction through financial management is significant and in a positive direction. The value is > 1.96 and the *p*-value is 0.000 less than 0.05. Therefore, the idea that financial attitude has an impact on financial satisfaction through financial management is acknowledged.

## Conclusion

Based on the findings of the data analysis, we discovered that financial management, through which financial attitude is mediated, has a positive and significant impact on financial happiness. This study demonstrates that the majority of respondents have excellent financial attitudes that allow them to deal with financial difficulties in a way that is in harmony with their financial management and results in their happiness with that management. Real socialization is required for the general population, particularly Generation Z, to spend money more responsibly in order to improve the quality of financial attitude, financial management, and financial satisfaction.

### Limitation, recommendation, and future research directions

Due to the limited time of the implementation of this research, the number of respondents obtained in this study is limited and may not reflect the population in this study. In order to achieve more reliable research results, it is necessary to take into account a larger population. We also suggest that the conceptual model be replicated and that further indicators be added to the study, such as parental income, self-confidence, locus of control, financial capacity, and impulsive behavior. We also recommend that future studies employ additional independent variables in addition to those used in this study, such as work and lifestyle. The number of individuals or samples that can generate reliable results is anticipated to increase with more research.

## Data Availability

Not applicable.
